# Effects of berberine and red yeast on proinflammatory cytokines IL-6 and TNF-α in peripheral blood mononuclear cells (PBMCs) of human subjects

**DOI:** 10.3389/fphar.2014.00230

**Published:** 2014-10-20

**Authors:** Carmen Spatuzza, Loredana Postiglione, Bianca Covelli, Margherita Ricciardone, Claudio Benvenuti, Paolo Mondola, Anna Belfiore

**Affiliations:** ^1^Unità di Fisiologia, Dipartimento di Medicina Clinica e Chirurgia, University “Federico II”Naples, Italy; ^2^Unità di Patologia Clinica, Dipartimento di Scienze Mediche Traslazionali, University “Federico II”Naples, Italy; ^3^Dipartimento Medico, Rottapharm MadausMonza, Italy

**Keywords:** interleukin 6, tumor necrosis factor α, berberine, red yeast, obesity, inflammation

## Abstract

**Background and Aims:** Obesity is a condition associated with chronic or acute inflammatory response characterized by an increase of proinflammatory cytokine levels. Peripheral blood mononuclear cells (PBMCs) migrate in adipose tissue inducing synthesis and secretion of adipocytokines as IL-6 and TNF-α. The aim of this study was to investigate the effect of berberine (a natural alkaloid) and red yeast (a natural antioxidant) on IL-6 and TNF-α cytokines release and gene expression, in circulating lipopolisaccarides (LPS) stimulated PBMCs.

**Methods and Results:** PBMCs isolated from whole blood of healthy donors were stimulated with LPS to induce cytokines production; simultaneously cells were treated with increasing doses of berberine and red yeast. The substances were administered alone or in association. IL-6 and TNF-α protein levels in the culture medium and their mRNA levels were assessed by ELISA and real time PCR, respectively. Berberine and red yeast treatment prevented the LPS induction of IL-6 release in the culture medium of PBMCs. In addition, berberine plus red yeast treatment showed a synergic inhibitory effect on IL-6 release at low concentration. Berberine and red yeast showed an inhibitory effect also on LPS induction of TNF-α release exerting a synergic effect mainly at high concentrations. On the contrary, berberine and red yeast did not significantly affect IL-6 and TNF-α mRNA levels induced by LPS. In this case, only concomitant treatment of PBMCs with high doses of berberine and red yeast inhibits LPS induced IL-6 or TNF-α mRNA levels.

**Conclusions:** The results of our study show that both berberine and red yeast were able to carry out anti-inflammatory action through an inhibition of proinflammatory IL-6 and TNF-α protein release. Moreover, when given in combination these substances were able to inhibit IL-6 and TNF-α gene expression in PBMCs activated by LPS. Therefore, these substances could represent a useful pharmacological treatment to reduce the proinflammatory status accompanied with obesity.

## Introduction

Many data indicate that obesity is associated with either a chronic or an acute inflammatory response characterized by an increase of cytokine production (Bastard et al., 2006). Recent data show that circulating mononuclear cells are involved in these increased proinflammatory proteins production in obese subjects. This effect seems to be mediated by the migration of mononuclear cells (MNCs) to arterial wall as well as into adipocyte tissue (Weisberg et al., 2003), in this tissue, MNCs induce adipocytokines such as interleukin 6 (IL-6) and tumor necrosis factor α (TNF-α) (Solá et al., 2009).

Some studies indicate that monocytes and macrophages are strongly increased in the adipose tissue of obese subjects where they produce proinflammatory cytokines that further increase the secretion of adipocyte proinflammatory proteins (adipokines) (Ghanim et al., 2004).

Berberine is an alkaloid extracted from plants belonging to Berberidaceae family that possesses many biological and pharmacological effects, such as inhibition of adipogenesis, anti-inflammatory activity, reduced insulin resistance, and potential immunomodulatory properties (Choi et al., 2006; Lee et al., 2006; Yi et al., 2008).

Red Yeast is the product of *Monascus purpureus* micetes rice fermentation showing strong antioxidant properties. Monacolin K, one of the red yeast metabolically active compounds, is a 3-hydroxy-3-methylglutaryl-coenzyme A reductase inhibitor effective in reducing the cholesterol synthesis (Lin et al., 2008a). This is a statin-like effect.

Even if several molecular mechanisms involved in the pathogenesis of chronic inflammation in obesity take place into adipose tissue, many studies demonstrate a MNCs involvement in generating proinflammatory cytokines overexpression in obese subjects (Ghanim et al., 2004). It is known that MNCs are able either to migrate through blood vessels in order to generate foam cells, contributing to atherosclerosis plaques formation, or to migrate in adipose tissue to produce proinflammatory cytokines like TNF-α and IL-6 (Weisberg et al., 2003). It is also well known that lipopolisaccarides (LPS) are recognized by TR4 membrane receptor mainly localized on monocytes/ macrophages membrane surface; the TR4 receptor activation carries out the production of many proinflammatory cytokines. Therefore, LPS stimulating MNCs may provide a representative model of the overall inflammatory status of body as well as of adipose tissue.

This study was aimed to investigate the effect of berberine and red yeast on proinflammatory cytokines, IL-6 and TNF-α protein release, and their expression in LPS stimulated PBMCs from donors buffy coats. Moreover, this study would further evaluate whether there is a synergism of these molecules in reducing proinflammatory cytokine levels.

## Materials and methods

### PBMCs extraction and stimulation

PBMCs were isolated from healthy donor buffy coats using Ficoll Paque gradient (GE Healthcare Europe, Munich, Germany). All samples were obtained in accordance with the requirements of the hospital ethics committee. The investigation conformed to the principles outlined in the Declaration of Helsinki (Rickham, 1966).

Briefly, donor blood was diluted 1:10 in PBS 1X and stratified on Ficoll solution with a 3:1 v/v ratio. After a 30 min centrifugation at 2200 r.p.m., PBMCs were recovered and re-suspended in RPMI-1640 medium supplemented with 10% FCS. 125000/mL PBMCs were cultured at 37°C in an humidified atmosphere with 5% CO_2_. MNCs were stimulated with 50 ng/mL LPS (*E. Coli* Lipopolisaccarides—Sigma-Aldrich, St. Louis, MO) to induce cytokines production. Simultaneously, cells were treated with increasing doses (1, 10 and 50 μg/mL) of Berberine and red yeast containing 1.5% S.C. (Specific Concentration) of the metabolic active compound Monacolin K, singularly or in association for 3, 6 or 24 h. Red yeast was kindly gifted by Rottapharm Spa. After incubation cells were recovered with cultured medium and centrifugated for 5 min at 2000 rpm. Supernatants containing secreted cytokines were collected.

### ELISA assay

TNF-α and IL-6 concentrations in diluted (1:100) culture supernatants were measured by enzyme linked immunosorbent assay (ELISA) according to instruction of the manufacturer (R&D Systems ELISA kit Minneapolis MN 55413, USA).

### RNA extraction

After PBMCs stimulation, cells were collected and total RNA was extracted by using the TRIzol reagent (Invitrogen, Karlsruhe, Germany). Extracted total RNA was quantified and the ratio A_260/280_ was measured to verify its purity.

### Real time PCR

0.5 μg of total RNA were reverse-transcribed (Invitrogen Life Technologies Ltd, Paisley, UK) and real time RT-PCR was performed using iCycler Apparatus (Bio-Rad, CA). Forty PCR amplification cycles of 60 s (15 s, 95°C; 45 s, 60°C) were run and amplification rates were monitored by the Sybr Green method. For PCR amplification the following primers were used: GAPDHf: GAA GGT GAA GGT CGG AGT C, GAPDHr: GAA GAT GGT GAT GGG ATT TC, TNF-αf: AAG AGT TCC CCA GGG ACC TCT, TNF-αr: CCT GGG AGT AGA TGA GGT ACA, IL-6f: GGA CGG CTT TTA CTT AAA CGC CAA GG, IL-6r: ATC TTC CCT AGT TAC CCA GGT TCA GC.

### Cell viability analysis

The cell viability, performed by Trypan blue test (Doyle and Griffiths, 1998), was higher than 90% in all the experimental conditions.

### Statistical analysis

Results were expressed as mean ± *SD*.

Kruskall-Wallis non parametric analysis of Variance followed by Mann-Whitney *U* test were used to compare the results.

## Results

### Berberine and red yeast (1.5% S.C. monacolin K) reduce proinflammatory IL-6 and TNF-α cytokines secretion in LPS activated PBMCs

**Figure 1:**
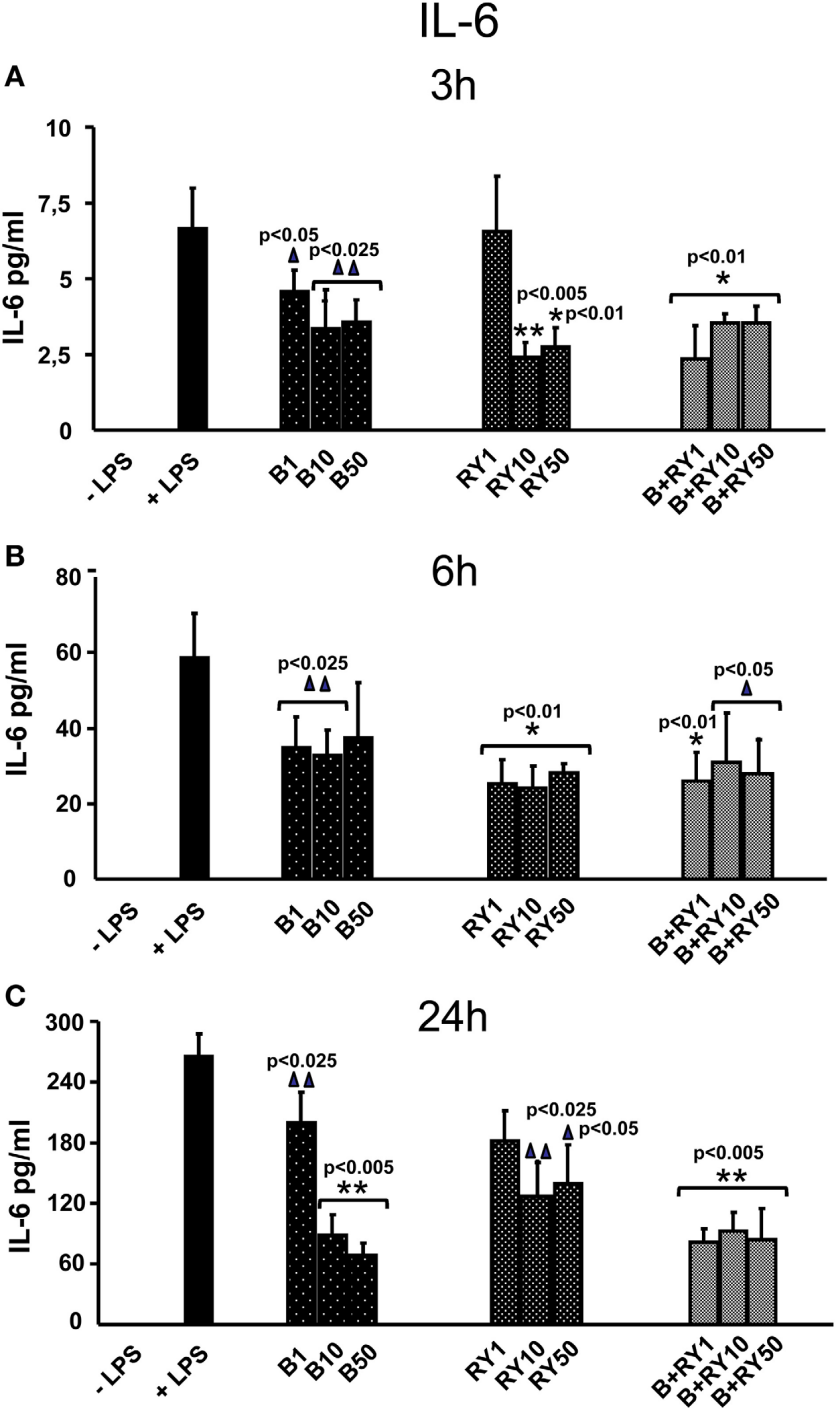
**Anti-inflammatory effects of berberine (B) at 1, 10 and 50 μg/mL (B1, B10, B50), red yeast (RY) at 1, 10 e 50 μg/mL (RY1, RY10, RY50) and berberine + red yeast at 1, 10 e 50 μg/mL (B+RY1, B+RY10, B+RY50) at 3 (A), 6 (B) and 24 h (C) after LPS stimulation (50 ng/mL) on IL-6 protein levels measured in the incubation medium by ELISA assay**. Asterisks indicate a significant value at Kruskal-Wallis non parametric analysis of variance followed by Mann-Whitney *U* test (^*^p < 0.05). Values are means ±*SD* of three experiments conducted in duplicate.

LPS treatment of PBMCs induced the secretion of proinflammatory IL-6 and TNF-α proteins. Berberine treatment reduced LPS inflammatory response by decreasing IL-6 release (Figure 1). The anti-inflammatory effect was significant at 3 and 24 h (*p* < 0.05) (Figures 1A,C). Equally, red yeast reduced inflammatory response lowering IL-6 release in LPS activated PBMCs (Figure 1). We observed an inhibition of IL-6 secretion after 3 h and 6 h of treatment with 10 and 50 μg/mL red yeast compared to LPS treated cells (*p* < 0.05) (Figures 1A,B). After 24 h of incubation, red yeast significantly decreased IL-6 release at all the doses used (*p* < 0.05) (Figure 1C). We observed that IL-6 secretion inhibition in berberine plus red yeast stimulated PBMCs cells was more evident at low concentration (1 μg/mL) and at short time (3 h) (65.2%) (*p* < 0.05). In the same conditions, berberine or red yeast alone inhibited IL-6 secretion by 33% and 3%, respectively (Figures 1A–C).

**Figure 2:**
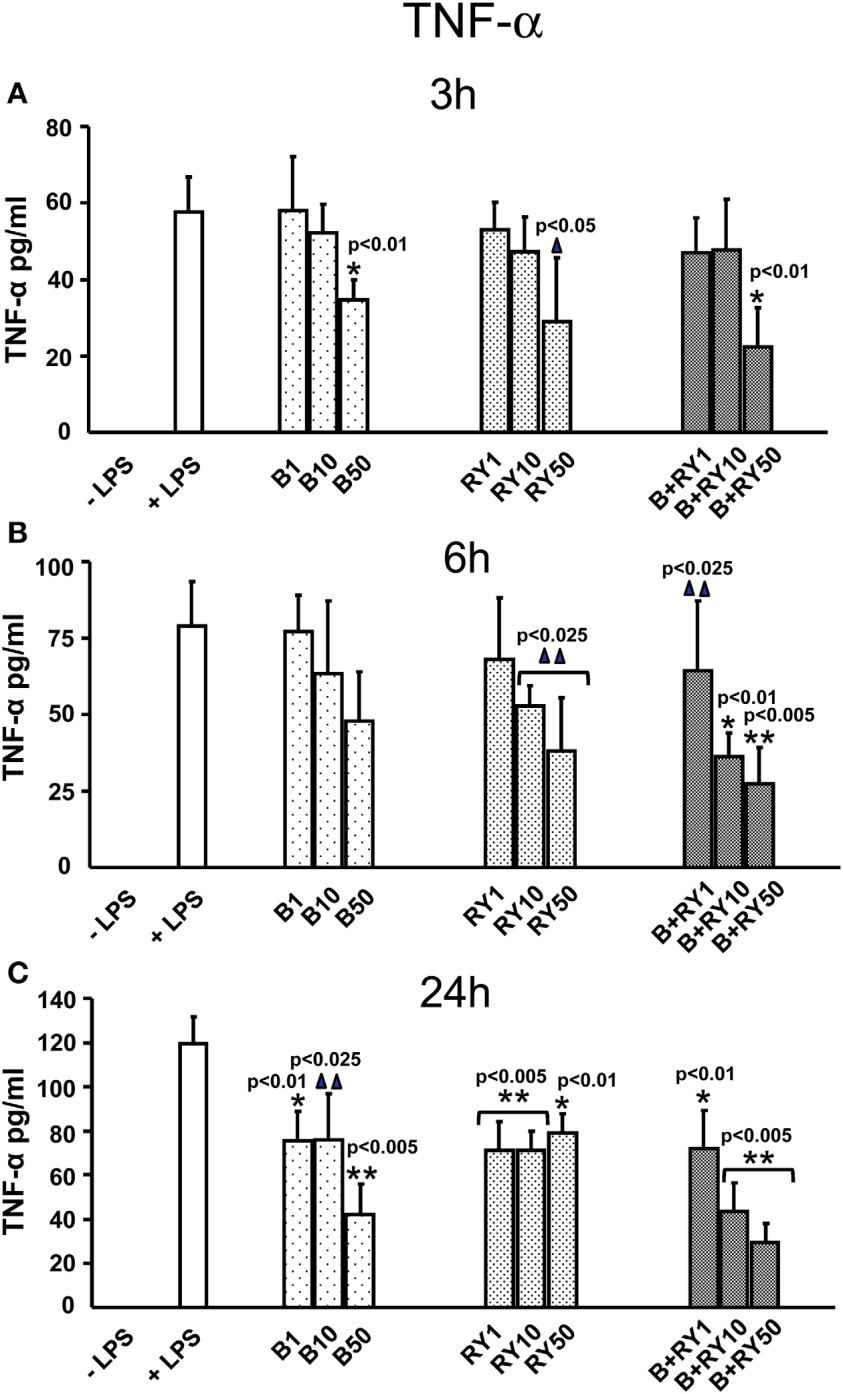
**Anti-inflammatory effects of berberine (B) at 1, 10 and 50 μg/mL (B1, B10, B50), red yeast (RY) at 1, 10 e 50 μg/mL (RY1, RY10, RY50) and berberine + red yeast at 1, 10 e 50 μg/mL (B+RY1, B+RY10, B+RY50) at 3 (A), 6 (B), and 24 h (C) after LPS stimulation (50 ng/mL) on TNF-α protein protein levels measured in the incubation medium by ELISA assay**. Asterisks indicate a significant value at Kruskal-Wallis non parametric analysis of variance followed by Mann-Whitney *U* test (^*^p < 0.05). Values are means ±*SD* of three experiments conducted in duplicate.

Berberine plus red yeast treatment carried out a synergic inhibitory effect on TNF-α secretion at all the incubation times; this effect was dose-dependent (Figures 2A–C). After 3 h of treatment the inhibition was equal to 61.5% with the concentration of 50 μg/mL (*p* < 0.05). After 6 h of incubation the synergic inhibitory effect was evident at 10 and 50 μg/mL (*p* < 0.05) (Figure 2B), while TNF-α release was maximally inhibited by 64.7% after 24 h at 50 μg/mL of berberine plus red yeast treatment, compared to LPS treatment (*p* < 0.05) (Figure 2C).

### Berberine and red yeast (1.5% S.C. monacolin K) reduce IL-6 and TNF-α mRNA levels in LPS activated PBMCs

Experiments of real time PCR were carried out starting from RNA of PBMCs stimulated with LPS. LPS treatment on PBMCs caused the induction of IL-6 and TNF-α mRNA levels.

**Figure 3:**
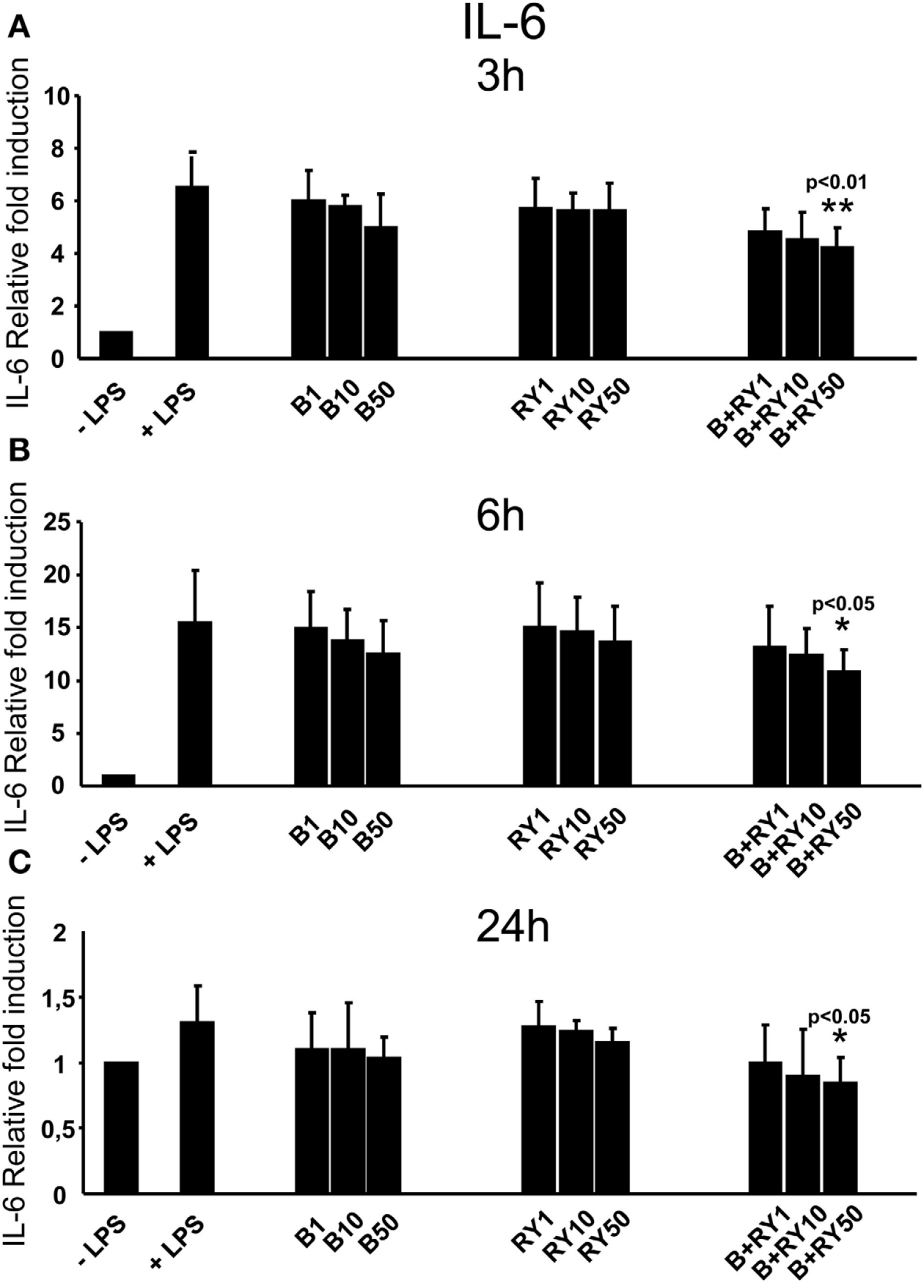
**Anti-inflammatory effects of berberine (B) at 1, 10 and 50 μg/mL (B1, B10, B50), red yeast (RY) at 1, 10 e 50 μg/mL (RY1, RY10, RY50) and berberine + red yeast at 1, 10 e 50 μg/mL (B+RY1, B+RY10, B+RY50) at 3 (A), 6 (B), and 24 h (C) after LPS stimulation (50 ng/mL) on IL-6 mRNA levels measured by RT-PCR**. Asterisks indicate a significant value at Kruskal-Wallis non parametric analysis of variance followed by Mann-Whitney *U* test (^*^p < 0.05). Values are means ±*SD* of three experiments conducted in duplicate.

**Figure 4:**
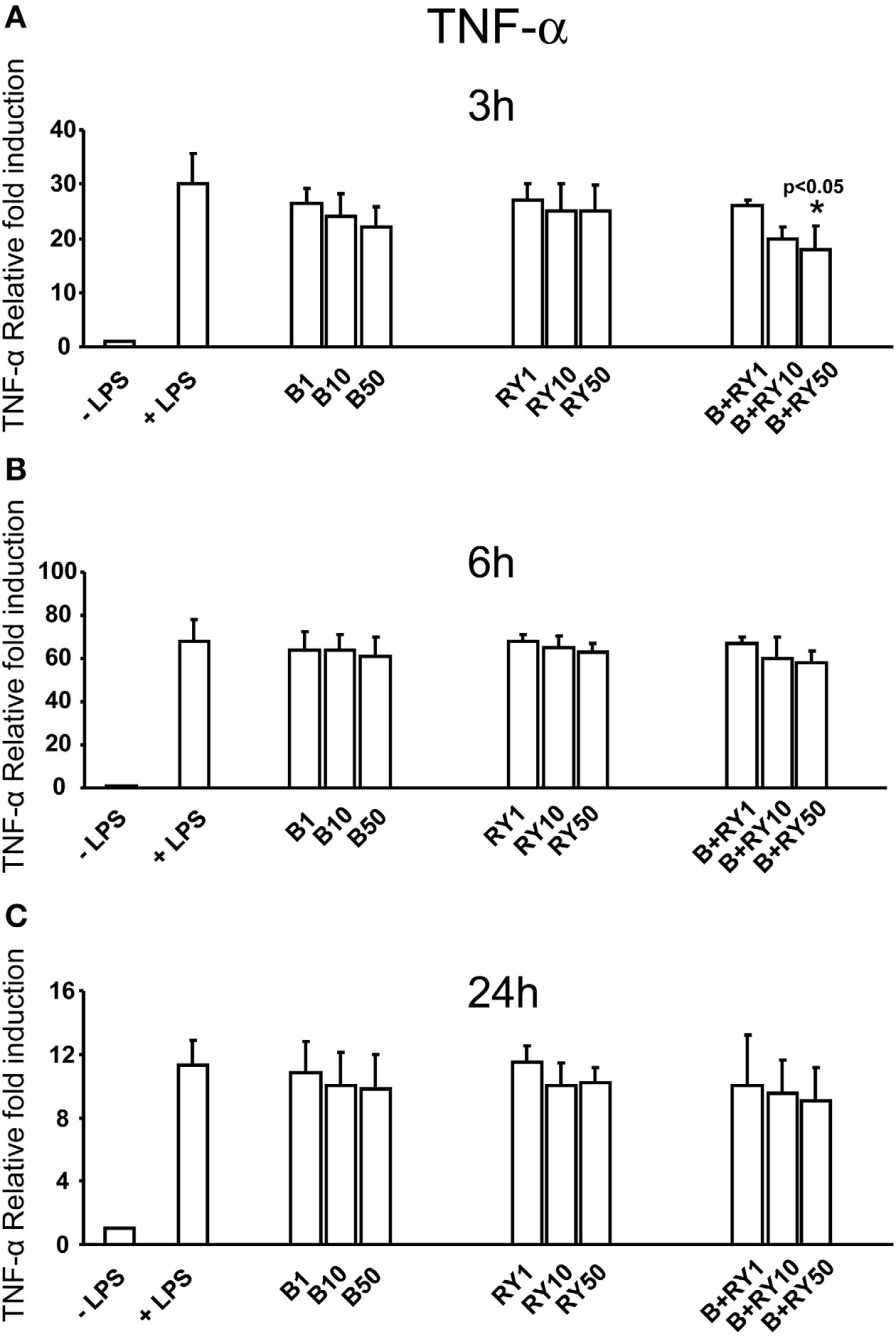
**Anti-inflammatory effects of berberine (B) at 1, 10 and 50 μg mg/mL (B1, B10, B50), red yeast (RY) at 1, 10 e 50 mg/mL (RY1, RY10, RY50) and berberine + red yeast at 1, 10 e 50 μg/mL (B+RY1, B+RY10, B+RY50) at 3 (A), 6 (B), and 24 h (C) after LPS stimulation (50 ng/mL) on TNF-α mRNA levels measured by RT-PCR**. Asterisks indicate a significant value (*p* < 0.05 at Kruskal-Wallis non parametric analysis of variance followed by Mann-Whitney *U* test). Values are means ±*SD* of three experiments conducted in duplicate.

Berberine determined a slight reduction of LPS induced IL-6 mRNA levels at 3, 6 and 24 h, that was not statistically significative (Figures 3A-C). At the same time, red yeast had not a significant inhibitory effect on IL-6 mRNA levels (Figures 3A–C). Differently, concomitant treatment with 50 μg/mL, of berberine and red yeast inhibited LPS induced IL-6 mRNA levels at 3, 6 and 24 h (*p* < 0.05) of treatment (Figures 3A–C). In addition, TNF-α inhibition was statistically significant with berberine and red yeast concomitant treatment at 3 h (*p* < 0.05), but not at 6 and 24 h (Figures 4A–C).

## Discussion

It has been previously demonstrated that mononuclear cells MNCs in obese subjects are involved in proinflammatory state with increased formation of proinflammatory transcriptional intranuclear factor kB (NF-kB) and proinflammatory genes regulated by NF-kB (Ghanim et al., 2004). Berberine anti-inflammatory effects have been largely demonstrated both in animal models and in other experimental systems (Ivanovska and Philipov, 1996). In addition, experimental evidence indicate a decrease of mRNA levels of inflammation marker (TNF-α, IL-6 and CRP) in berberine-treated immortalized 3T3-L1 adipocytes (Choi et al., 2006). In the last years, moreover, different studies have demonstrated red yeast anti-inflammatory effects (Chen et al., 2008; Lin et al., 2008b).

The results of our study show that both berberine and red yeast carried out anti-inflammatory action through an inhibition of proinflammatory cytokines, IL-6 and TNF-α release and their gene expression in PBMCs activated by LPS. It is worth noting that berberine and red yeast were active in a synergistic way; we observed that IL-6 secretion inhibition in berberine plus red yeast stimulated PBMCs cells was more evident at low concentration (1 μg/mL) and at short time (3 h) (65.2%). In the same conditions, berberine or red yeast alone inhibited IL-6 secretion by 33% and 3%, respectively. Synergistic effect of berberine and red yeast was also evident in TNF-α secretion inhibition; in this case this synergism appreciated during entire time-course (3, 6 e 24 h), but at high concentration (50 μg/mL). Berberine plus red yeast were effective in a synergistic way also during cytokines transcription or post-transcription events regulating mRNA levels. It is evident from our data that, except for the combination of both compounds, there is no effect of both substances administered alone on mRNA levels of TNF-α or IL-6. Nevertheless, this suggest that both substances carried out an effect at post-transcriptional level even if this hypothesis needs further investigation.

Recent studies have shown berberine protective effect in the treatment of metabolic disorders such as body weight reduction, cholesterol, and glycemia level reduction (Lee et al., 2006; Yin et al., 2008; Affuso et al., 2010). In addition, several studies attribute to berberine an anti-obesity effect, because it is able to inhibit adipogenesis (Choi et al., 2006; Huang et al., 2006; Kim et al., 2007; Yi et al., 2008; Hu and Davies, 2010). It has been reported that monacolin K is a red yeast metabolically active compound effective in plasma lipid lowering (Cicero et al., 2010). According to previous data (Bastard et al., 2000; Juge-Aubry et al., 2004; Aygun et al., 2005; Park et al., 2005), our results indicate that the induction of proinflammatory cytokines, IL-6 and TNF-α production, caused in PBMs stimulated by LPS, is similar to that observed in obese subject, where metabolic disorders, proinflammatory state and insulin resistance are often associated. Therefore, our study suggests that berberine and red yeast could represent a useful pharmacological approach to reduce proinflammatory status associated with these pathologies.

### Conflict of interest statement

The authors declare that the research was conducted in the absence of any commercial or financial relationships that could be construed as a potential conflict of interest.
