# Supplementary figures and images for: Effects of berberine and red yeast on proinflammatory cytokines IL-6 and TNF-α in peripheral blood mononuclear cells (PBMCs) of human subjects
Source: Front Pharmacol. 2014 Oct 20;5:230. doi: 10.3389/fphar.2014.00230 (PMC4202723; doi:10.3389/fphar.2014.00230)

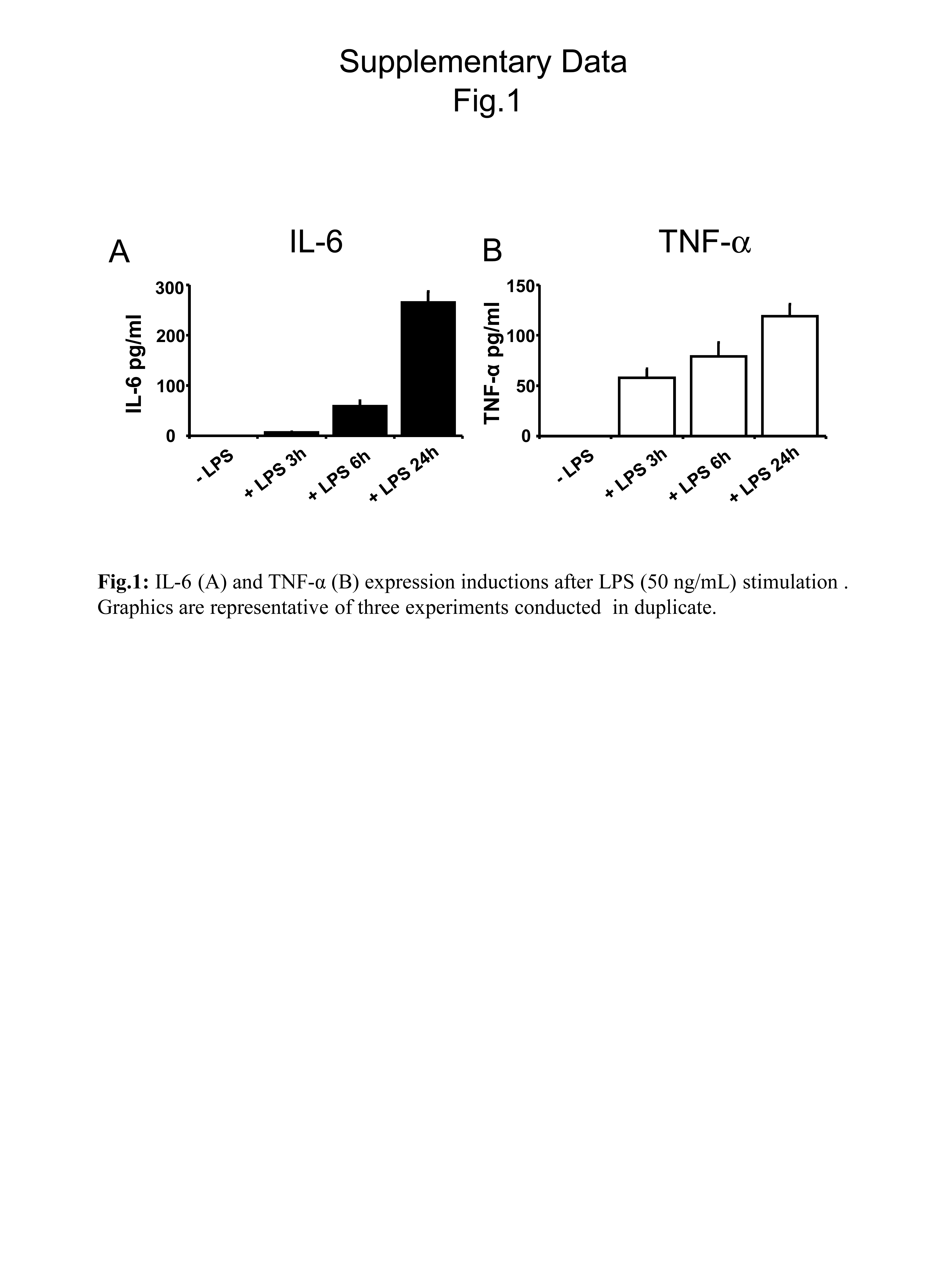

Supplement: Supplementary file 1 [file Image1.TIF]

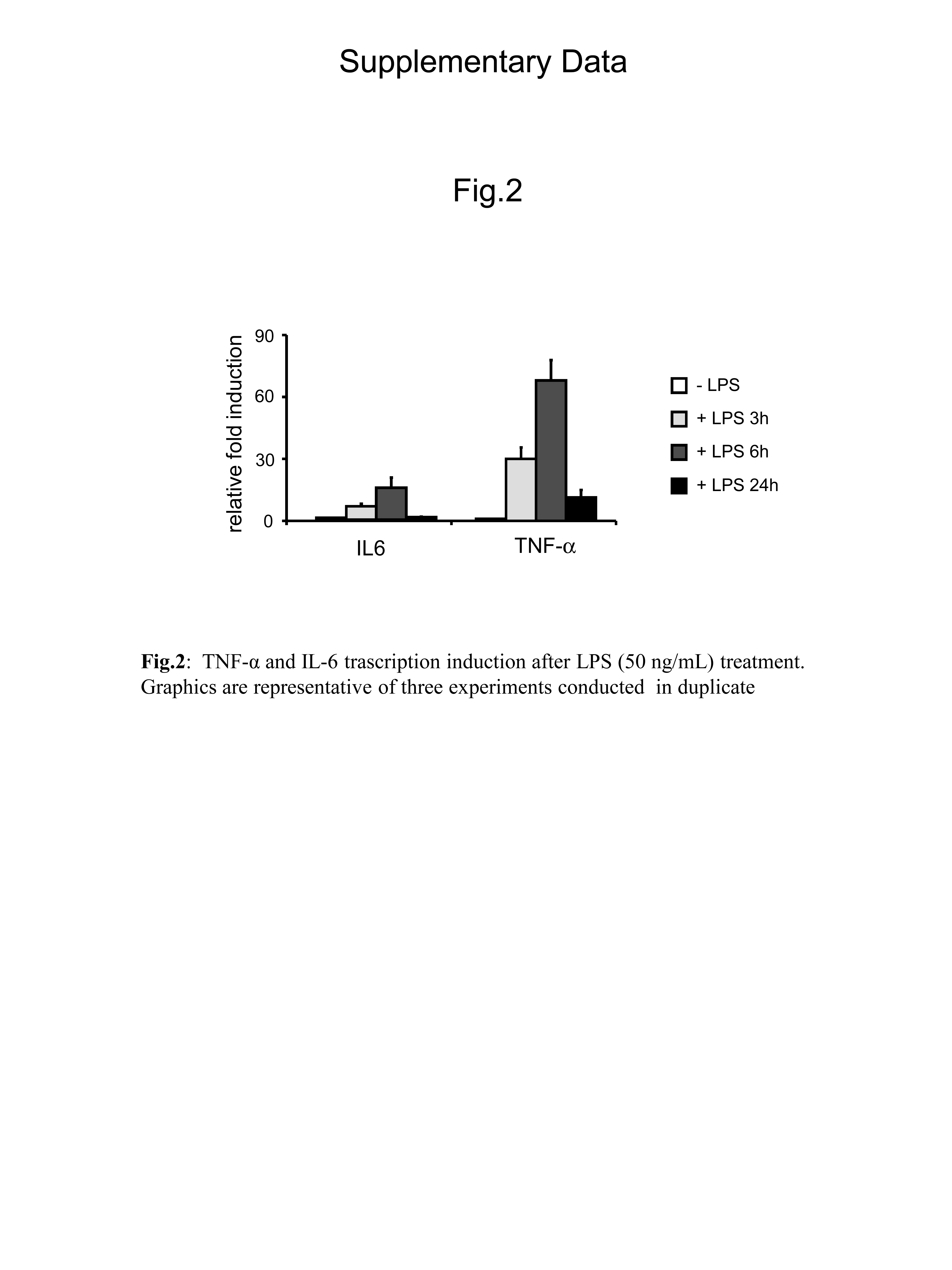

Supplement: Supplementary file 2 [file Image2.TIF]
